# Development and Application of a Pragmatic Algorithm to Guide Definitive Carbapenemase Testing to Identify Carbapenemase-Producing *Pseudomonas aeruginosa*

**DOI:** 10.3390/antibiotics9110738

**Published:** 2020-10-27

**Authors:** Christian M. Gill, Tomefa E. Asempa, David P. Nicolau

**Affiliations:** 1 Center for Anti-Infective Research and Development, Hartford Hospital, Hartford, CT 06102, USA; christian.gill@hhchealth.org (C.M.G.); Tomefa.Asempa@hhchealth.org (T.E.A.); 2 Division of Infectious Diseases, Hartford Hospital, Hartford, CT 06102, USA

**Keywords:** carbapenemases, *Pseudomonas aeruginosa*, molecular diagnostics, susceptibility testing, phenotypic carbapenemase testing, antimicrobial susceptibility testing

## Abstract

A minimum inhibitory concentration (MIC) derived algorithm, predictive of carbapenemase production, was developed using a challenge set (*n* = 92) of *Pseudomonas aeruginosa* (PA), including carbapenemase-producing (CP), cephalosporinase and/or efflux/porin mutation, and wild-type isolates. Broth microdilution MICs to clinically relevant anti-pseudomonal agents were utilized. The algorithm was applied to 1209 clinical PA isolates from a US surveillance program. Confirmatory genotypic (Xpert^®^ Carba-R assay) and phenotypic (mCIM/eCIM) testing for carbapenemases was conducted on algorithm-derived isolates. With the algorithm, carbapenem resistance alone resulted in poor specificity to identify CP-PA (54%) within the challenge set of isolates. Inclusion of cefepime, ceftazidime, and piperacillin/tazobactam non-susceptibility resulted in a specificity of 66%. Ceftolozane/tazobactam resistance further improved specificity (89%). Of the 1209 isolates, 116 met criteria (carbapenem-resistant and non-susceptibility to cefepime, ceftazidime, and piperacillin/tazobactam) for confirmatory testing. Carba-R and mCIM/eCIM identified five (all *bla*_VIM_-positive) and seven carbapenemase-producing isolates, respectively. This MIC algorithm combined with genotypic/phenotypic carbapenemase testing is a pragmatic and streamlined approach to identify CP-PA.

## 1. Introduction

Carbapenem-resistance among *Pseudomonas aeruginosa* is mediated by several resistance mechanisms, including drug efflux, porin loss, inducible AmpC, and carbapenemase activity, resulting in difficult-to-treat infections [1,2,3,4]. The introduction of new β-lactam–β-lactamase inhibitor combinations has improved the management of carbapenem-resistant *P. aeruginosa* (CRPA); however, certain enzymatic resistance mechanisms still remain a challenge [5,6]. 

Globally, carbapenemase-producing *P. aeruginosa* prevalence and diversity is largely based on geography [1]. For example, 20% of doripenem-non-susceptible *P. aeruginosa* from Europe harbored carbapenemase genes compared with 77% of carbapenem-resistant *P. aeruginosa* in certain regions of Latin America [7,8]. A recent report from the US National Healthcare Safety Network highlighted approximately 2% of CRPA harbored carbapenemases [3]. Similarly, 4.3% of CRPA in a Canadian surveillance study were carbapenemase producers [9]. Carbapenemase production among *P. aeruginosa* is problematic as carbapenemase genes tend to be located on transmissible genetic material. This is especially troubling as many frequently encountered carbapenemases, such as some Verona integron-encoded metallo-β-lactamase (VIM) harboring clones, are plasmid mediated, which can result in rapid spread, even in the absence of antibiotic selective pressure [10,11]. Locally, detection of carbapenemase-producing CRPA impacts infection control and therapeutic strategies as these infections are associated with nosocomial spread, limited treatment options, and increased mortality [12,13,14].

Several tests to detect carbapenemase activity have been evaluated; however, they can be laborious and/or have slower turn-around time (i.e., phenotypic tests such as modified carbapenem inactivation method (mCIM) or CarbaNP) or are cost-prohibitive (i.e., genotypic testing) [15]. The majority of screening algorithms aimed at increasing the likelihood of detecting carbapenemase production among organisms have been evaluated in *Enterobacterales* [16,17,18] but are limited for *P. aeruginosa* [19]. A pragmatic algorithm using antimicrobial susceptibility results readily available in the clinical laboratory may help identify *P. aeruginosa* isolates most likely to produce carbapenemases to optimize laboratory time and resources.

The purpose of this study was to (1) develop an algorithm that would streamline the use of confirmatory carbapenemase detection methodologies and (2) apply this algorithm to clinical *P. aeruginosa* isolates from a US surveillance study.

## 2. Results

### 2.1. Algorithm Development

From the challenge set (Table S1), imipenem and meropenem resistance alone poorly differentiated carbapenemase-producing from non-carbapenemase-producing *P. aeruginosa* isolates (sensitivity: 100%, 95%CI 94–100%; specificity: 54%, 95%CI 37–71%). All carbapenemase-producing isolates were resistant to ceftolozane/tazobactam while, as expected, ceftazidime/avibactam resistance was carbapenemase class dependent. Table 1 describes the sensitivity and specificity of different antimicrobial susceptibility testing criteria when applied to the challenge set. 

**Figure 1:**
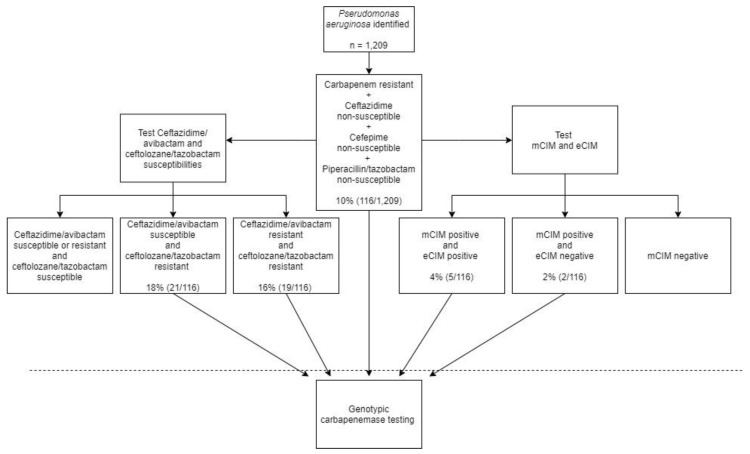
Algorithm for carbapenemase detection in *Pseudomonas aeruginosa*. The percent (number of isolates meeting current criteria/ number meeting preceding criteria) refers to application of the algorithm to the surveillance program evaluated. mCIM, modified-carbapenem inactivation method; eCIM, EDTA-modified carbapenem inactivation method.

All carbapenemase-positive isolates demonstrated resistance to carbapenems and non-susceptibility to cefepime, ceftazidime, and piperacillin/tazobactam (sensitivity: 100%, 95%CI 94–100%; specificity: 66%, 95%CI 48–81%). Surprisingly, applying stricter criteria of resistance to all agents (i.e., carbapenems, cefepime, ceftazidime, piperacillin/tazobactam) failed to capture 10 carbapenemase-producing isolates from the challenge set, resulting in a decrease in sensitivity (sensitivity: 83%, 95%CI 70–91%), with all 10 isolates testing intermediate to piperacillin/tazobactam. Inclusion of ceftolozane/tazobactam resistance to the carbapenem-resistant and non-susceptibility to cefepime, ceftazidime, and piperacillin/tazobactam criteria increased specificity without compromising sensitivity (specificity: 89%, 95%CI 73–97%). Adding ceftazidime/avibactam resistance further increased specificity (91%) but compromised sensitivity (86%) because the challenge set included *Klebsiella pneumoniae* carbapenemase (KPC)-harboring isolates to which ceftazidime/avibactam was susceptible. In an effort to develop a pragmatic screening algorithm that can be easily adopted, a final susceptibility criterion of carbapenem-resistant, cefepime-, ceftazidime-, and piperacillin/tazobactam-non-susceptible was selected for further clinical application given that ceftolozane/tazobactam and ceftazidime/avibactam susceptibility testing may not be universally available. Figure 1 depicts the antimicrobial susceptibility derived-algorithm for carbapenemase screening of *P. aeruginosa*.

### 2.2. Application of Algorithm against Clinical P. aeruginosa Isolates 

From the 1209 clinical isolates from the US surveillance study, 230 (19%) were imipenem and meropenem resistant. Application of the screening algorithm resulted in 116 (10%) *P. aeruginosa* isolates meeting criteria for higher probability of carbapenemase production (Table 2). As a result, all 116 isolates underwent confirmatory genotypic and phenotypic testing for evidence of carbapenemase production.

Genotypic testing with Xpert^®^ Carba-R revealed five isolates (4%) that harbored carbapenemases. All isolates were VIM-positive and were initially submitted to the surveillance study from four different medical centers. The five VIM-harboring isolates were resistant to cefepime, ceftazidime, meropenem, and imipenem while non-susceptible to piperacillin/tazobactam. 

Phenotypic testing with mCIM identified positive carbapenemase production among seven isolates (6%). Furthermore, the EDTA-modified carbapenem inactivation method (eCIM) test classified five of these seven isolates as producing metallo-dependent enzymes, concordant with genotypically identified VIM-harboring isolates. Whole-genome sequencing of the two mCIM-positive, eCIM-negative isolates demonstrated evidence of a *bla*_GES-20_ gene in one isolate, a previously documented carbapenemase [20]. The second mCIM-positive strain harbored *bla*_OXA-2_, *bla*_OXA-50_, and PAO. Notably neither OXA-2 nor OXA-50 enzymes are thought to be carbapenemases but together may contribute to antimicrobial resistance or the isolate contains a carbapenemase that is outside of the known database. Based on genotypic and phenotypic testing, seven isolates were categorized as carbapenemase-producing in this collection of *P. aeruginosa* from US medical centers.

## 3. Discussion

The incidence of CRPA is on the rise, and currently, genotypic or phenotypic confirmation of carbapenemase production is recommended to initiate infection control measures [21]. Given the heterogeneous resistance mechanisms harbored by *P. aeruginosa*, we sought to develop a practical screening algorithm using susceptibility testing to streamline genotypic and phenotypic carbapenemase testing for the identification of carbapenemase production among clinical *P. aeruginosa* isolates, especially as carbapenem resistance in *P. aeruginosa* in the United States is largely driven by alterations in oprD [22]. Genotypic and phenotypic carbapenemase testing methods identified five and seven *P. aeruginosa* isolates with evidence of carbapenemase activity respectively, from an algorithm-derived group of isolates from US centers (*n* = 116). 

All five metallo-β-lactamase producing CRPA identified in our study harbored the *bla*_VIM_ gene. This finding is concordant with previous reports where *bla*_VIM_ are the most common carbapenemase genotypes among *P. aeruginosa* reported in the US [3,23]. Two additional isolates identified by the algorithm tested positive on mCIM (indicative of carbapenemase enzyme production), negative on eCIM (indicative of the absence of a metallo-dependent enzyme), and negative on Carba-R. This phenotype tends to describe enzyme subtypes not identified with current Carba-R probe targets, and subsequent whole-genome sequencing (WGS) testing detected one isolate harboring OXA-2, OXA-50, and PAO, while the second harbored a GES-20 carbapenemase. The first isolate lacked any enzymology consistent with known carbapenemases, which may indicate additive resistance mechanisms can produce false-positive mCIM results. This finding was similar to what has been noted by Simner and colleagues; in their multicenter analysis, an isolate with the same genotype tested mCIM positive at 8 of the 10 testing sites [24]. The Guiana-Extended-Spectrum β-lactamase (GES)-harboring isolate, a known carbapenemase, may represent a growing clinical challenge. The prevalence of GES-producing *P. aeruginosa* varies by geography, and while several reports from Mexico and Canada have been published, there are limited data evaluating this enzymology in the US [9,25,26]. Furthermore, *bla*_GES_ is not a genotypic target on any of the three current FDA-approved platforms [27,28,29]. Based on our findings from this US surveillance study, GES enzymes may be an underappreciated contributor to carbapenem resistance among *P. aeruginosa* but can be characterized by pairing the developed algorithm with mCIM. However, the developed algorithm will still be a useful starting point to guide definitive carbapenemase testing using future iterations of molecular diagnostics or other validated phenotypic screens. 

The current algorithm was designed to be a simple tool that incorporates routinely utilized anti-pseudomonal β-lactam susceptibilities and increases the likelihood of detecting carbapenemase production with definitive carbapenemase testing given that resistance to carbapenems alone is not evidence of carbapenemase activity in *P. aeruginosa* [4]. Of note, aztreonam was not included in the phenotypic algorithm as it may not be routinely reported by laboratories, and although it is stable to metallo-β-lactamase degradation, it is labile to co-expressed Extended-Spectrum β-lactamases (ESBLs) and cephalosporinases [30]. A global surveillance study found only 25% of isolates harboring Class B enzymes test as aztreonam-susceptible [31]. Incorporation of susceptibility testing to the newer antimicrobial agents, i.e., ceftolozane/tazobactam and ceftazidime/avibactam, to the algorithm provides further specificity. Based on our challenge set, and other published data [6,32], most carbapenemases render ceftolozane/tazobactam resistant, while ceftazidime/avibactam resistance is carbapenemase class dependent. Depending on local epidemiology of carbapenemase classes (i.e., high prevalence of KPC-producers), addition of ceftazidime/avibactam resistance may decrease the sensitivity of testing criteria. Nonetheless, addition of both agents aids in risk-stratifying isolates for carbapenemase detection testing, significantly improving test sensitivity and specificity. Additionally, both ceftolozane/tazobactam and ceftazidime/avibactam have therapeutic utility against carbapenem-resistant *P. aeruginosa* and thus warrant susceptibility testing [2,33]. Unfortunately, despite ceftolozane/tazobactam and ceftazidime/avibactam being available on commercially utilized automated susceptibility testing systems in the United States, both are categorized as selective reporting by Clinical and Laboratory Standards Institute (CLSI) [34]. Furthermore, some institutions utilize reflex testing criteria, which may lead to delays in testing and reporting. Hence, our algorithm was based on routinely reported agents (i.e., ceftazidime, cefepime, and piperacillin/tazobactam) for use when ceftolozane/tazobactam and ceftazidime/avibactam testing is delayed or unavailable. Newer agents such as imipenem-relebactam and meropenem-vaborbactam may be useful for carbapenemase-detection screening and warrant further evaluation [2,35]. Notably, the addition of vaborbactam offers no advantage to meropenem alone for *P. aeruginosa* [35].

How frequently a microbiology laboratory performs genotypic and phenotypic testing to detect carbapenemase-producing organisms will depend on local prevalence rates. However, given the rising incidence of carbapenem-resistant *P. aeruginosa* isolates, prioritizing isolates to undergo confirmatory carbapenemase testing is essential to avoid unnecessary utilization of laboratory resources. Previous testing algorithms to identify carbapenemase-producing organisms have generally focused on *Enterobacterales.* In addition, prior algorithms involved specialized testing methods not routinely used in the clinical laboratory (i.e., temocillin disks) [16,17,18]. When considering *P. aeruginosa*, Samuelson and colleagues coupled ceftazidime minimum inhibitory concentration (MIC) ≥ 8, imipenem MICs > 8, and four phenotypic methods limited to metallo-β-lactamase detection only [19]. Sixty-two carbapenem-resistant *P. aeruginosa* isolates were tested, and two metallo-β-lactamase producing isolates were detected [19]. Notably, the addition of ceftazidime and imipenem susceptibility criteria decreased the number of tests run and increased the positive predicted value of each phenotypic test evaluated [19]. A recent publication described the implementation of a carbapenemase testing algorithm including *P. aeruginosa*. The authors utilized non-susceptibility to meropenem and non-susceptibility to either ceftazidime or cefepime to cascade ceftolozane/tazobactam and ceftazidime/avibactam MIC testing with or without genotypic carbapenemase testing, depending on non-susceptibility to both agents [36]. Our data add to this approach; in our challenge set we found implementation of genotypic testing upon resistance to ceftolozane/tazobactam resulted in high sensitivity. Additionally, our data suggest non-susceptibility to cefepime, ceftazidime, and piperacillin/tazobactam may be a reasonable starting point to initiate genotypic testing while awaiting ceftolozane/tazobactam and ceftazidime/avibactam MICs to hasten infection control measures. The different testing criteria presented in our data provide clinicians and clinical laboratories options for implementing criteria-driven carbapenemase testing to meet the organization’s needs and priorities (i.e., broader testing with quicker result vs. stricter testing with longer time to result). 

A strength of this study is the algorithm derivation from a challenge set of *P. aeruginosa* isolates harboring diverse genotypic profiles, allowing application in regions with various carbapenem prevalence rates. Importantly, the sensitivity and specificities of each MIC testing criteria are predicated on the robustness of the challenge set, and subsequent test performance, specifically positive and negative predictive values, will vary in different clinical settings. This algorithm was designed as a starting point for prioritizing carbapenemases detection workflows for *P. aeruginosa* isolates, and local validation using local standards for MIC testing is warranted. 

In conclusion, the application of an algorithm incorporating conventional susceptibility testing can be utilized to stratify *P. aeruginosa* isolates to undergo additional genotypic and phenotypic carbapenemase detection methods. As the prevalence and diversity of carbapenemase-producing *P. aeruginosa* expands across the globe, adoption of screening algorithms in tandem with molecular testing will provide tremendous value in characterizing resistance mechanisms.

## 4. Materials and Methods 

### 4.1. Algorithm Development 

#### 4.1.1. Bacterial Isolates 

A challenge set of 92 *P. aeruginosa* isolates displaying diverse genotypic profiles: New Delhi metallo-β-lactamase (NDM) (*n* = 10); imipenemase (IMP) (*n* = 10); Verona integron-encoded metallo-β-lactamase (VIM) (*n* = 10); *Klebsiella pneumoniae* carbapenemase (KPC) (*n* = 8); Sao Paulo metallo-β-lactamase (SPM) (*n* = 10); Guiana-Extended-Spectrum β-lactamase (GES) (*n* = 9), meropenem or imipenem-resistant strains with known genotypic cephalosporinase or efflux/porin mutation (*n* = 20), and wild-type isolates (*n =* 15), was evaluated. Genotypic profiles were previously determined by PCR or whole-genome sequencing. Isolates were obtained from the CDC-FDA Antimicrobial Resistance Bank (Atlanta, GA, USA) and the Center for Anti-Infective Research and Development isolate repository.

#### 4.1.2. Antimicrobial Susceptibility Testing

Broth microdilution tests were performed on each *P. aeruginosa* isolate from the challenge set in concordance with CLSI standards [34]. The antimicrobial agents tested were obtained as laboratory-grade powders. All broth microdilution trays were prepared, and MIC testing was conducted at the Center for Anti-Infective Research and Development. Minimum inhibitory concentrations (MICs) to cefepime, ceftazidime, piperacillin/tazobactam, meropenem, imipenem, ceftolozane/tazobactam, and ceftazidime/avibactam were obtained and interpreted per CLSI standards [34]. 

### 4.2. US Surveillance Study

#### 4.2.1. Organism Collection

A total of 1209 *P. aeruginosa* isolates were submitted from 36 medical centers from around the United States to be utilized in a national antimicrobial susceptibility surveillance study (2016–2017) [37]. Approval was obtained from all sites or a waiver was obtained from local institutional review boards. Patients had to be at least 18 years old for study inclusion and isolate submission. These clinical *P. aeruginosa* isolates were prospectively collected from blood and respiratory samples and identified by the microbiology laboratory of each participating hospital via automated systems including VITEK^®^ (bioMérieux), BD Phoenix^TM^ Automated Microbiology System (Becton Dickinson), MicroScan^®^ (Beckman Coulter), and Matrix-Assisted Laser Desorption Ionization Time-of-Flight (MALDI-TOF) (VITEK^®^ MS Healthcare, bioMérieux) [37]. Antimicrobial susceptibility testing was conducted at the Center for Anti-Infective Research and development by broth microdilution as described above. 

#### 4.2.2. Phenotypic and Genotypic Carbapenemase Testing

Based on the antimicrobial susceptibility testing results of the challenge cohort, isolates that were carbapenem (meropenem and imipenem) resistant plus cefepime, ceftazidime, and piperacillin/tazobactam non-susceptible per CLSI guidelines [34] were selected for confirmatory phenotypic (mCIM/eCIM) and genotypic testing.

The modified carbapenem inactivation method (mCIM) and the EDTA-modified carbapenem inactivation method (eCIM) were conducted on each isolate as previously described [24,34,38]. Results were interpreted for mCIM and eCIM testing as defined previously by measuring the diameter of the zone of inhibition around each disk [34,38]. Quality control isolates included two negative controls (*K. pneumonia* ATCC 1706 and *P. aeruginosa* ATCC 27853), one serine-carbapenemase control (*K. pneumoniae* ATCC 1705, KPC positive), and a metallo-β-lactamase positive control (*K. pneumoniae* CDC Bank #505, NDM-positive). 

The Xpert^®^ Carba-R assay (Cepheid, Sunnyvale, CA, USA) was utilized to detect the presence of five common carbapenemase enzyme genotypes (*bla*_NDM,_ *bla*_IMP,_ *bla*_VIM,_ *bla*_KPC,_ and *bla*_OXA-48 like_). Testing was conducted per device package insert although subcultures were not performed in the presence of meropenem disks. Quality control assessments were conducted once-weekly during testing using the Xpert^®^ Carba-R QC Panel M219 (Main Molecular Quality Control, Saco, ME, Lot: D03JAN19B and E03JAN19B). 

Any isolate with discordant results between mCIM/eCIM and Carba-R assay underwent whole-genome sequencing (WGS). WGS was performed as follows: Each *P. aeruginosa* isolate was grown overnight prior to nucleic acid extraction using Qiagen DNeasy Blood and Tissue Kit (Qiagen, Valencia, CA, USA), and DNA concentrations were quantified using the NanoPhotometer system (Implen, Munich, Germany). Sequencing libraries were then then prepared using Nextera XT (Illumina, San Diego, CA) kit, quantified with the Qubit 4 Fluorometer using the dsDNA High Sensitivity Assay Kit (Invitrogen, Carlsbad, CA, USA), and finally sequenced with the Illumina MiSeq (Illumina, San Diego, CA, USA) sequencer. All testing was in compliance with manufacturer instructions. The resulting genomes were analyzed using the Center for Genomic Epidemiology (CGE) ResFinder online tools (https://cge.cbs.dtu.dk/services/ResFinder/) [39].

### 4.3. Statistical Analysis

The sensitivity and specificity values with their respective 95% confidence intervals (CI) to predict carbapenemase-production were calculated using IBM SPSS Version 23 (Armonk, NY).

## Figures and Tables

**Table 1:** Characteristics of the challenge set of 92 *P. aeruginosa* isolates utilized in algorithm development.

Susceptibility	Carbapenemase Producers, *n* = 57	Non-Carbapenemase Producers,		Test Performance	
		Cephalosporinase or Efflux/Porin Mutation, *n* = 20	Wild Type, *n* = 15	Sensitivity, % (95% CI)	Specificity, % (95% CI)
IPM + MEM- Resistant	57 (100%)	15 (75%)	1 (7%)	100% (94–100%)	54% (37–71%)
IPM + MEM- Resistant AND FEP + CAZ + TZP- Non-Susceptible	57 (100%)	12 (60%)	0 (0%)	100% (94–100%)	66% (48–81%)
IPM + MEM- Resistant AND FEP + CAZ + TZP- Resistant	47 (82%)	6 (30%)	0 (0%)	83% (70–91%)	83% (66–93%)
IPM + MEM- Resistant AND FEP + CAZ + TZP- Non-Susceptible + CZA- Resistant	49 (86%)	8 (40%)	0 (0%)	86% (74–94%)	77% (60–90%)
IPM + MEM- Resistant AND FEP + CAZ + TZP- Non-Susceptible + C/T- Resistant	57 (100%)	4 (20%)	0 (0%)	100% (94–100%)	89% (73–97%)
IPM + MEM- Resistant AND FEP + CAZ + TZP- Non-Susceptible + C/T- Resistant + CZA- Resistant	49 (86%)	3 (15%)	0 (0%)	86% (74–94%)	91% (77–98%)

IPM = imipenem; MEM = meropenem; FEP = cefepime; CAZ = ceftazidime; TZP = piperacillin/tazobactam; CZA = ceftazidime/avibactam; C/T = ceftolozane/tazobactam; Sensitivity and Specificity = calculated based on prediction the criteria accurately identify carbapenemase production; 95%CI = 95% confidence interval.

**Table 2:** Performance of algorithm after application to 1209 clinical *P. aeruginosa* isolates from a US surveillance study.

Algorithm-Derived Screening Criteria	Number Meeting Criteria	Carbapenemase Producers Detected	Carbapenemase Producers Missed by Criteria
IPM + MEM- Resistant AND FEP + CAZ + TZP- Non-Susceptible	116	7/116	0
IPM + MEM- Resistant AND FEP + CAZ + TZP- Non-Susceptible + CZA- Resistant	43	7/43	0
IPM + MEM- Resistant AND FEP + CAZ + TZP- Non-Susceptible + C/T- Resistant	21	6/21	1 *
IPM + MEM- Resistant AND FEP + CAZ + TZP- Non-Susceptible + C/T- Resistant + CZA-Resistant	19	6/19	1 *

IPM = imipenem; MEM = meropenem; FEP = cefepime; CAZ = ceftazidime; TZP = piperacillin/tazobactam; CZA = ceftazidime/avibactam; C/T = ceftolozane/tazobactam. * Genotype: *bla*_OXA-2_, *bla*_OXA-50_, and PAO.

## Supplementary Materials

The following are available online at https://www.mdpi.com/2079-6382/9/11/738/s1, Table S1: *P. aeruginosa* Challenge Set.
